# Brain-behaviour correlates of habitual motivation in chronic back pain

**DOI:** 10.1038/s41598-020-67386-8

**Published:** 2020-07-06

**Authors:** Frauke Nees, Michaela Ruttorf, Xaver Fuchs, Mariela Rance, Nicole Beyer

**Affiliations:** 1 Department of Cognitive and Clinical Neuroscience, Central Institute of Mental Health, Medical Faculty Mannheim, Heidelberg University, J5, 68159 Mannheim, Germany; 2 Computer Assisted Clinical Medicine, Medical Faculty Mannheim, Heidelberg University, Mannheim, Germany; 3 Department of Radiology and Biomedical Imaging, Yale University, New Haven, CT USA; 4 Institute of Medical Psychology and Medical Sociology, University Medical Center Schleswig-Holstein, Kiel University, Kiel, Germany

**Keywords:** Cognitive neuroscience, Learning and memory, Motivation, Reward, Diseases

## Abstract

Chronic pain may sap the motivation for positive events and stimuli. This may lead to a negative behavioural cycle reducing the establishment of appetitive habitual engagement. One potential mechanism for this might be biased learning. In our experiment, chronic back pain patients and healthy controls completed an appetitive Pavlovian-instrumental transfer procedure. We examined participants` behaviour and brain activity and reported pain, depression and anxiety. Patients showed reduced habitual behaviour and increased responses in the hippocampus than controls. This behavioural bias was related to motivational value and reflected in the updating of brain activity in prefrontal–striatal–limbic circuits. Moreover, this was influenced by pain symptom duration, depression and anxiety (explained variance: up to 50.7%). Together, findings identify brain-behaviour pathways for maladaptive habitual learning and motivation in chronic back pain, which helps explaining why chronic pain can be resistant to change, and where clinical characteristics are significant modulators.

## Introduction

Chronic pain is a burden for both the individual and the society representing major clinical, social and economic problems (e.g.,^1^), which can have a strong impact on the patients` quality of life. Using biomedical interventions or psychological treatments, pain and its negative consequences can be minimized. However, through a negative pain-reinforcing cycle, chronic pain patients mostly develop maladaptive pain managing behaviour, which is in conflict with these clinical interventions (e.g.,^2^).

This may be due to a sap in motivation for positive events and stimuli. Using an operant approach/avoidance task in animals,^3^ showed that under pain, animals have a significant reduction in approaching appetitive reward (to satisfy hunger). Moreover, the motivation to avoid pain superseded the motivation to alleviate hunger^3^. Although assessing the nature of chronic pain in humans and its maintenance have improved, in particular in the aversive domain, recent assessments mostly failed to examine the underlying motivational drives related to appetitive reward (for a review see e.g.^4^).

While in healthy individuals positive stimuli such as pleasurable food were shown to reduce acute pain perception^5^, chronic pain patients might benefit less from these positive stimuli and events, but rather focus on pain-related aspects of relief^6,7^. This is also indicated by findings that showed that (chronic) pain is associated with the inhibition of behavioural responses to obtain a reward (e.g.,^8^).

One potential mechanism for this reduction of appetitive habitual engagement might be biased by learning (e.g.,^9,10^), including the interaction of Pavlovian and instrumental learning processes. Such interactions are represented in Pavlovian-instrumental transfer (PIT): if an appetitive Pavlovian (conditioned) stimulus (CS) has been associated with a positive reinforcer, such as food, the CS can strongly enhance a positive behavioural response to this reinforcer (called instrumental responding), when the reinforcer is presented unexpectedly (PIT effect,e.g.,^11^). Pavlovian influences may lead to a general inhibitory or excitatory bias on instrumental responding. Pavlovian-conditioned responses may be evolutionarily hard wired and thus explicitly linked to incentive, motivational, valence^12^, and this is an important dimension in the modulation of cognitively controlled behaviour^13–15^.

PIT effects have been shown to depend on the mesolimbic dopaminergic system, with brain regions like the striatum, prefrontal cortex, amygdala, hippocampus being involved (e.g.,^16^). In pain, activation in these regions have been shown to mediate contextual and affective aspects of pain processing (e.g.,^5,17–19^). Moreover, they might also come into play when acute pain turns into chronic pain (e.g.,^20^), and thus represent significant contributors to pain pathophysiology (e.g.,^9,21–24^).

In our study we therefore examined chronic back pain patients completing a PIT task during functional magnetic resonance imaging (fMRI) and compared them to control individuals. We also integrated information on reported pain, depression and anxiety as crucial clinical characteristics that can change an individuals` behaviour (e.g.,^10,25^) as well as reduce motivation and anhedonia (e.g.,^26–31^).

## Results

### Brain-behaviour correlates of PIT in chronic back pain patients versus healthy controls

**Figure 1:**
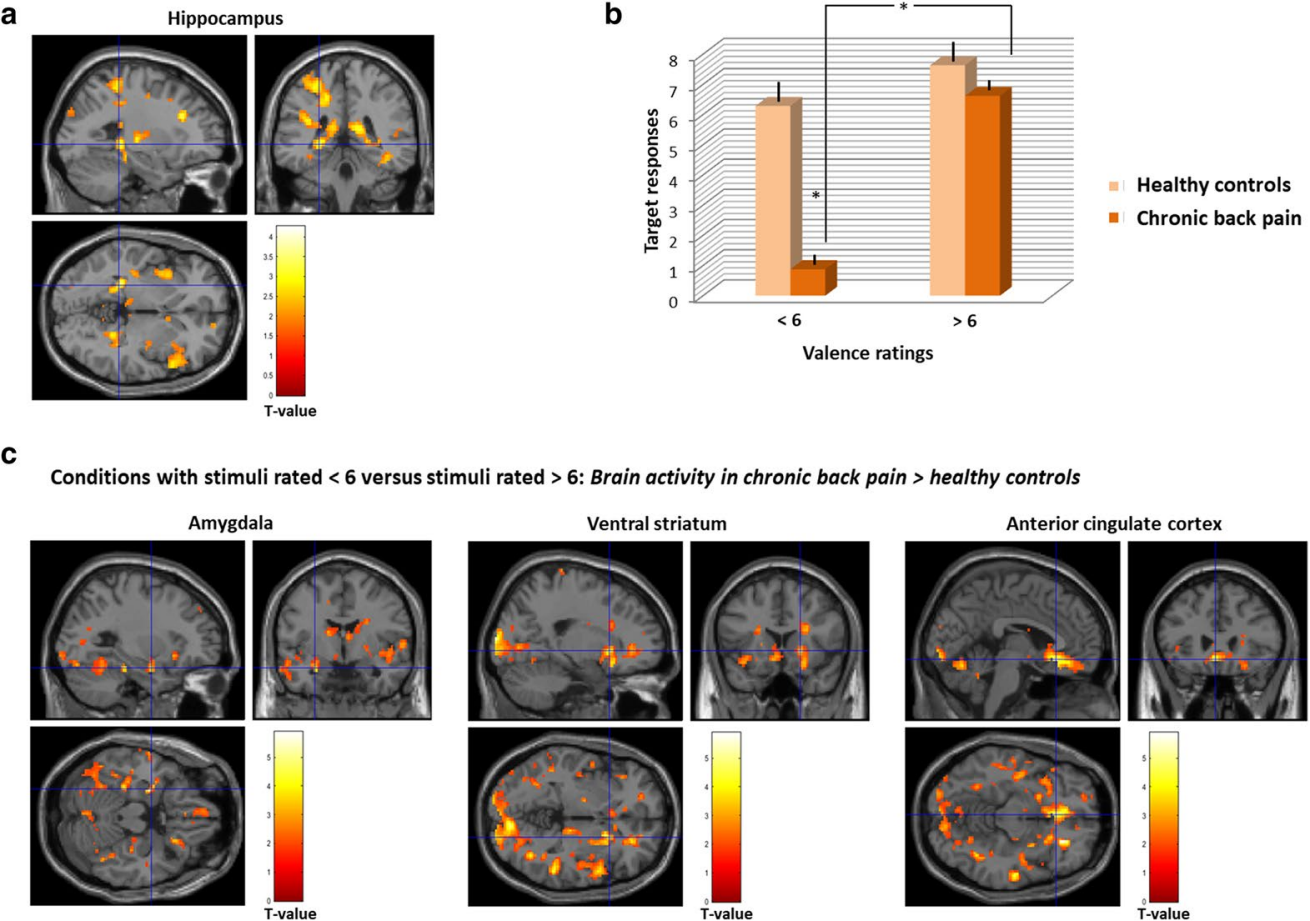
(**a)** Increased response in the hippocampus during Pavlovian-instrumental transfer in chronic back pain patients versus healthy controls (family-wise error rate corrected (FWE) < 0.05, peak-level)); (**b)** Behavioral responses to conditioned stimuli (CSs) during PIT and valence ratings to these CSs in back pain patients compared to controls; (**c)** Brain responses during PIT to CSs with higher versus lower valence in chronic back pain patients compared to controls (family-wise error rate corrected (FWE) < 0.05, peak-level).

During PIT, patients showed reduced transfer behaviour compared to controls (F(1,59) = 4.963; *p* = 0.023) and increased responses in the hippocampus (x =  − 25, y =  − 38, z =  − 2; k = 77; t = 3.67; *p* = 0.041; Fig. 1. No significant main effect of group was found for other brain regions of interest (ROIs) like the insula, amygdala, PFC, VS and ACC.

### Motivational value

Chronic back pain patients compared to controls showed a stronger decrease in PIT behaviour when CSs were rated with lower valence (valence score of 5–6; F(1,59) = 33.082, *p* = 0.031; behavioural responses to stimuli of lower versus higher (score > 6) valence scores: (t(7) =  − 7.977; *p* < 0.001; Fig. 1b. Brain responses to higher versus lower rated CSs were significantly increased in the VS (x = 21, y = 17, z =  − 2, k = 67; t = 5.30; *p* = 0.048) and the amygdala (x =  − 28, y =  − 4, z =  − 20; k = 27; t = 4.17; *p* = 0.036) and reduced response in the ACC (x =  − 7, y = 33, z = 16; k = 21; t = 4.87; *p* = 0.047) in patients compared to control Fig. 1c (see also Figure S1 in the Supplements).

### Impact of pain symptom duration on brain correlates of PIT and the role of depression and anxiety

**Figure 2:**
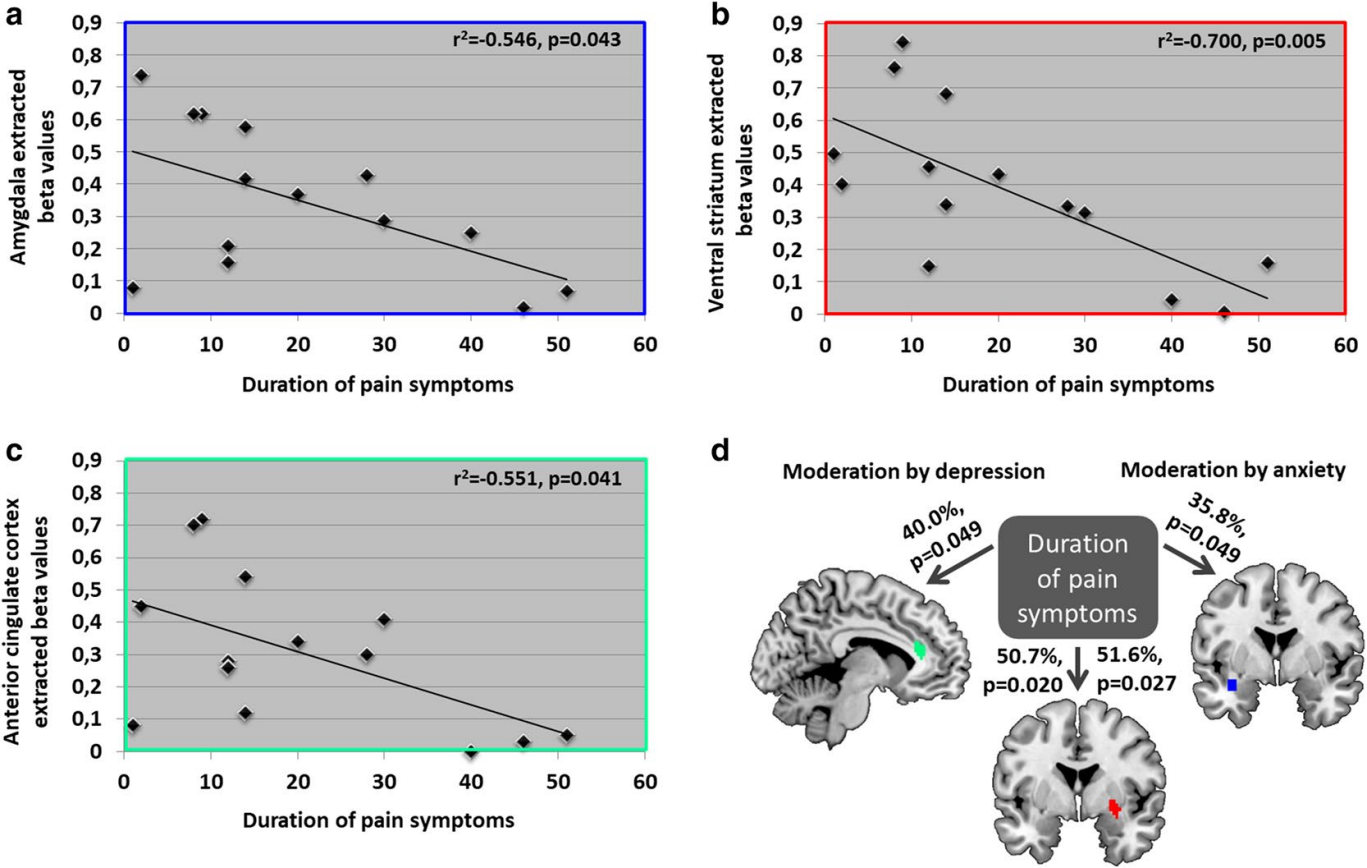
Correlation between brain responses in (**a)** the amygdala, (**b)** ventral striatum and (**c)** anterior cingulate cortex during Pavlovian-instrumental transfer and the duration of pain symptoms in chronic back pain patients, and (**d)** their moderations by anxiety and depression. *Note* N = 8 patients did not provide any information on the duration of their pain symptoms, which resulted in N = 22 patients for these analyses.

In the chronic back pain patients, we tested whether the duration of pain was directly associated with the observed brain changes during PIT: pain duration was significantly negatively correlated with amygdala (r =  − 0.553, *p* = 0.043; Fig. 2a, VS (r =  − 0.706, *p* = 0.005; Fig. 2b and ACC (r =  − 0.548, *p* = 0.041, Fig. 2c responses (i.e. the longer the duration of pain symptoms the reduced the brain responses), with significant modulation by depression for the ACC (*p* = 0.049, F(2,20) = 3.343, explained variance: 40%) and the VS (*p* = 0.027, F(2,20) = 5.236, explained variance: 51.6%), and by anxiety for the amygdala (*p* = 0.049, F(2,21) = 3.162, explained variance: 35.8%) and the VS (*p* = 0.020, F(2,21) = 5.563, explained variance: 50.7%, Fig. 2d.

## Discussion

After repeated experiences, many of our initially voluntary behaviours can become habitual to allow a reflexive and automatic process. This process can be adaptive initiating positive approach or negative avoidance behaviour, and may be based upon learning mechanisms, where Pavlovian CSs energize instrumental behaviour (PIT effect). For chronic pain, voluntary behaviour is focused on pain and pain-related stimuli and this might occur at the cost of focusing on positive events and stimuli. PIT to positive stimuli may therefore be reduced in chronic pain (e.g.,^32^).

In our study, we found that chronic back pain patients exhibited reduced behavioural PIT responses, together with increased brain responses in the hippocampus. In previous studies on PIT, increased hippocampus activity has been suggested to relate to stronger learning-related psychomotor, behavioural, activity^16^. In our patients, we observed an opposite pattern in that increased responses in the hippocampus were associated with a failure to integrate learned appetitive responses into respective behaviour. Aside its integration in psychomotor activity, hippocampus activation triggers memory process and encodes of (spatio)configural, contextual or relational information from the environment (e.g.,^33,34^), and was found to relate to pain intensity in the context of imagined painful situations^35^. Patients might get stuck in such memory processes and thus were hindered in any transfer of this memory into behaviour. These memory processes might here not only relate to the newly learned PIT associations induced by the PIT procedure, but also to their previous experiences, particularly to constantly predominant pain, due to an overactive contextual embedding of learned associations. This could hinder any proper encoding of the acquired positive cued associations relevant for the motivational state of the patients due to a general cognitive over-commitment to pain and in consequence reduced approach towards pleasurable events and stimuli, which has been observed previously (e.g.,^5–8^). We identified Pavlovian-instrumental learning as one of the possibly critical mechanisms. Future studies should integrate pain-related stimuli in PIT procedures to specifically target and manipulate the interaction of aversive and appetitive aspects.

Animal studies on chronic pain also discuss a role of hyperexcitability of the hippocampus being a possible chain for alterations in synaptic plasticity in the nucleus accumbens^36^. In our study, we also observed differences in striatal–limbic–prefrontal responses during PIT in back pain patients compared to controls. Responses in the VS, amygdala and ACC were related to valence ratings of the CSs, which was also found to determine respective PIT behaviour. Although both patients and controls rated the CSs as highly valent, patients showed a strong significant decrease in behavioural responses when valuation was under a specific threshold. So, there may not only be a mismatch or dysfunctional interrelation between a motivational drive to and changes in responding to pleasure and hedonic aspects in chronic pain^5^, but patients seem to need a much higher degree and/or amount of rewarding stimulation, which might often fail in daily live, and could explain the observed reduced motivational drive to positive aspects. The promotion of (instrumental) action selection to approach positive outcomes and the strong staggered relation to the value of these outcomes may thus represent a key component of maladaptive behaviour in chronic back pain. In this context, the striatum, and here foremost ventral parts, may transfer this valuation and encode the associated processes including tendencies towards related behaviour^37^. This may include a control of moment-by-moment choices (e.g.,^38,39^). Moreover, activation in the amygdala, together with responses in the ACC, might cause a corresponding reduced emotional embedding and a reduction in performance control to gain pleasure, which has also previously been found to be present only at very high levels of stimulus valence (e.g.,^8^).

Interestingly, brain responses in the VS, amygdala and ACC were also significantly associated with the duration of pain symptoms in the patients. This relates to previous data that have described a shift from activating sensorimotor brain regions in acute pain to emotion-related circuitry following pain chronification^20^. Moreover, we have also determined the role of depression and anxiety (e.g.,^10,25^), as they have been related to reduced motivation and anhedonia and shown to be associated with pain processing and chronicity (e.g.,^26–31^). In the patients, we found that depression and anxiety significantly modulated the association between pain symptom duration and brain responses during PIT (for the ACC modulated by depression, for the amygdala by anxiety, and for the VS by both depression and anxiety). Although patients did not show significantly higher levels of depression than the controls, these findings might still speak for a significant role of mood under chronic pain conditions. Patients with a longer history of pain might be more sensitive to even smaller changes in depression. This adds for example also to findings on a significant role of the ACC for depressive symptoms in chronic pain and as important target underlying mechanisms of pain sensations^40^.

Although chronic back pain has often been reported to be more prevalent in females (e.g.^41,42^), our sample consisted to a large proportion of male patients. This might have co-determined our effects, as male sex have been shown a positive association with improvement in pain disability^43^, however, other studies also demonstrated no significant association between disability and sex^44,45^. For experimental pain, greater pain sensitivity has been reported among females than males for most pain modalities, evidence regarding sex differences in endogenous pain modulation, measured in the laboratory, yet, are mixed, as are findings on fMRI to ascertain differences in pain-related brain responsivity in females compared with males (for a review see^46^). With respect to our current findings, we might speculate that effects might have even been stronger in samples that comprised a larger proportion of females, but that the involved brain areas might overlap.

Together, our findings form the present study provide further insight into learning and motivational processes in chronic back pain, that could explain the often observed maladaptive coping behaviour. Moreover, there might be subgroups within the population of chronic back pain patients depending on the affective symptom spectrum of depression and anxiety. Our data can be used to understand individual differences in behavioural change and can inform longitudinal studies to further address the role of emotional learning in the development of chronic pain. They can also be used as informative targets in the therapeutic field to improve motivation for and adherence to chronic pain interventions. Interventions may include strategies to re-learn focusing on pleasurable stimuli and events, which can also help to reduce their focus on pain(-related) stimuli, and the role of comorbid affectivity should be more specifically integrated in pain therapies^21,47^. Moreover, in future studies, an investigation of the functions of the dopaminergic system and its interaction with the opioid system in this context might provide further interesting information on the regulation of motivated behaviour^48,49^, reward-prediction errors in instrumental learning and vigor in appetitive PIT^50^ in chronic pain.

## Methods

### Participants

We investigated primary chronic lower or upper back pain patients (N = 30; mean age = 53; 9 females) and healthy controls (N = 30; mean age = 47; 7 females), who did not differ between age, sex and education (see Table 1 for sample description).

**Table 1:** Characteristics of the study samples.

	Back pain patients	Controls	*p* value
Number	30	30	–
Age, years; mean (SD)	53 (± 13.21)	47 (± 15.48)	n.s
Sex female; number	9	7	–
Formal education, years; median (range)	12.53 (8–18)	13.21 (8–17)	n.s
Anxiety; mean (range)	40.32 (± 13.41)	29.8 (± 6.56)	< 0.01
Depression; mean (range)	14.18 (± 11.55)	8.7 (± 7.25)	n.s
Pain experience scale			
*Affective*	27.36 (± 7.89)	–	
*Sensory*	18.71 (± 6.62)	–	
*Sensory—rhythmicity*	5.29 (± 2.64)	–	
*Sensory—penetration*	8.57 (± 3.25)	–	
*Sensory—temperature*	4.86 (± 2.14)	–	
Multidimensional pain inventory			
*Pain intensity*	3.23 (± 0.96)	–	
*Impairment*	3.49 (± 1.07)	–	
*Affective mood*	2.45 (± 0.83)	–	
*Social support*	2.48 (± 1.36)	–	
*Control of life*	4.29 (± 1.39)	–	
*Punishment*	0.53 (± 0.87)	–	
*Attention*	1.85 (± 0.8)	–	
*Distraction*	2.25 (± 1.46)	–	
*Social activities*	2.55 (± 1.29)	–	
*Activities at home*	3.88 (± 1.17)	–	
*Activities outdoor*	1.67 (± 1.33)	–	
*Total score*	8.1 (± 2)	–	

*SD* standard deviation, *n.s.* non-significant.

As treatment recommendations for chronic pain patients strongly indicate continuous pharmacotherapy, we did not per se exclude patients with psychotropic medication, but defined a medication-free period of at least 4 weeks prior to investigation as a prerequisite. Previous medication and dose of medication were carefully assessed and used as covariate in subsequent analyses.

With respect to the PIT task (description see below), participants who did not rate the valence of the CSs positively (below a score of five) following the Pavlovian conditioning phase were not included in the present study to avoid alterations in the PIT effect due to non-learning of association as negative/aversive.

Exclusion criteria were: chronic and current substance abuse, any neurological disease, left-handedness, major illness, pregnancy, a pacemaker or metal parts in the body, and for the patients additionally pain after traumatic experiences/accidents, pain surgery, pain due to physical decline. Inclusion criteria for the patients were: pain localized to the upper or lower back, with a minimum pain intensity of four on a 0–10 point scale that should occur minimum three times/week (and is classified as interfering), and lasts for more than 6 months of pain.

The study was approved by the local Ethics committee from the Medical Faculty Mannheim, Heidelberg University. Informed consent was obtained from all participants, and all methods were carried out in accordance to the relevant guidelines and regulations.

### Psychometric assessment

All participants completed the Structured Clinical Interview for DSM-IV (SCID-I;^51,52^), the State-Trait Anxiety Scale^53^, the General Depression Scale^54^, a structured pain interview^55^, the West Haven-Yale Multidimensional Pain Inventory^56^ and the Pain Experience Scale^57^. For the analyses of the present study, we used depression and anxiety mean scores as well as years of symptom duration.

### Appetitive Pavlovian-instrumental transfer task

All participants underwent a PIT task (e.g.,^58,59^, see Figure S2 in the Supplements) during functional magnetic resonance imaging (fMRI).

As unconditioned stimuli (USs), we used three food pictures and conditioned stimuli (CSs) were four neutral fractal pictures, three of them paired with a food picture and one of them presented without any pairing. Before the experiment, participants had to rate different food pictures on a pleasantness scale to select their preferred food. We used the most highly and equally valued food items and individuals were told that they get the food on the picture at the end of the experiment, if they show correct button presses.

The task consisted of three phases: instrumental conditioning, in which participants were instructed to press one of three buttons located under the three food pictures (USs), followed by Pavlovian conditioning, in which participants were instructed to watch four different fractal pictures (CSs), three of them were presented together with the previously used food pictures from the instrumental phase, one as presented alone, and the PIT phase, in which participants were instructed to press a button when the fractal pictures from the Pavlovian phase were presented.

#### Instrumental conditioning phase

During the instrumental trials (duration: 6 s each), two of three squares at the bottom of the screen changed color from black to gray and participants were instructed to press a button that corresponds to one of the two gray squares and then one of the food pictures was presented on the left side of the screen.

#### Pavlovian conditioning phase

In the Pavlovian trials (duration: 6 s each), the CSs (fractal pictures) were presented on the right side of the screen, and the food pictures from the previous instrumental phase were displayed again on the left side of the screen (each of the three food pictures were linked to one specific fractal (CS+) throughout the phase, one fractal picture (CS−) was presented without linkage to a food picture).

#### PIT phase

During the PIT trials (duration: 6 s each), participants were presented a gray square on the left side of the screen (at the location where the food pictures were displayed during the previous phases) and the CSs were presented at the right side of the screen. Similar to the instrumental conditioning phase, two of three squares at the bottom of the screen changed color from black to gray and participants were instructed to press a button that corresponds to one of the two gray squares. The stimuli were presented in the following trial combinations: button one and button two presented with CS one, button one and button two presented with CS two, button two and button three presented with CS two, button one and button three presented with CS three and button two and button three presented with CS three. This constellation allowed testing for specific PIT effects (e.g.,^59^). During instrumental and Pavlovian conditioning each trial type was presented 15 times, during PIT 20 times.

#### Subjective ratings

Valence ratings of the USs and CSs (food and fractal pictures) were assessed after each of the three phases using the Self-Assessment Manikin (^60^) that was transferred to a 1–9 scale (1 = very unpleasant to 9 = very pleasant).

### Magnetic resonance imaging

We performed magnetic resonance imaging in a 3 T Tim TRIO whole body scanner (SIEMENS Healthineers, Erlangen, Germany) using a 12-channel head coil. To account for maximum magnetic field homogeneity we did shimming and we recorded a standard gradient field map before starting the task-based fMRI sequence. For this functional protocol, we applied the following parameters using a T_2_*-weighted gradient-echo echo-planar imaging (EPI) sequence with GRAPPA technique and 40 contiguous axial slices: slice thickness of 2.3 mm, slice gap of 0.7 mm, descending slice order, acceleration factor 2, repetition time (TR) of 2350 ms, echo time (TE) of 22 ms, matrix size of 96 × 96, field of view (FoV) of 220 × 220 mm^2^, flip angle (α) of 90°, bandwidth (BW) of 1270 Hz/px. We further obtained a T_1_-weighted magnetization prepared rapid gradient echo (MPRage) sequence for structural reference, with 192 sagittal slices and the following parameters: TR of 2300 ms, TE of 2.98 ms, matrix size of 240 × 256, field of view (FoV) of 240 × 256 mm^2^, flip angle (α) of 9°, bandwidth (BW) of 240 Hz/px).

### Data (pre)processing and statistical evaluation

#### fMRI data

For fMRI data analyses we used Statistical Parametric Mapping software (SPM12 (v6685), Wellcome Trust Centre for Neuroimaging, Institute of Neurology, University College London, UK), implemented on MATLAB R2016a (The MathWorks Inc., Natick, MA, USA). We excluded the first three scans from our analyses, and preformed a gradient field map correction on the remaining EPI images. This was followed by realignement to the fourth image using a rigid body transformation, creation of a mean image, and correction of the realigned and unwarped images for differences in acquisition time. A coregistration of the mean image to the T_1_ structural image was then applied, and we normalized the anatomical image into a standard stereotactic space (MNI—Montreal Neurological Institute, Quebec, Canada). Data were smoothed (7 mm^3^ (full width half maximum) Gaussian kernel), and event-related blood-oxygenation level dependent (BOLD) responses were convolved with a canonical hemodynamic response function, a high-pass filter with a temporal cut-off of 128 s, and first-order autoregressive functions AR(1) to correct for serial autocorrelations.

All three phases of the task were modeled separately, but for the present study we focused on the PIT phase. During this phase, subject-specific regressors at the time of cue onset were included for the following conditions: the three CSs with a response option for the respectively learned associated food (CS+), the non-food-related stimulus (CS−) and six scan-to-scan motion parameters regressors of no interest. In a second level random effects analysis, we subsequently included the individual contrast images using the full factorial model of SPM12, and performed a non-sphericity correction to account for the problem of non-independent data within subjects and error variance heterogeneity. Fixed effect analyses were then calculated for each subject and, in second level random effects analyses, two-sample *t* tests were performed for the contrast CS+ versus CS− . We further used a regression analysis in both pain patients and controls with valence ratings of the conditioned stimuli as covariate of interest to test for the affective components of the PIT effect. To ensure that effects were not co-determined by the learning performance during the instrumental and Pavlovian transfer, we used the behavioural responses during both phases (reaction rates and valence ratings) as covariates of no interest in our analyses. For all fMRI data analyses, we applied small volume correction at the voxel-level. Given the established literature highlighted in the introduction, we were interested in responses in the insula, amygdala, hippocampus, prefrontal cortex (PFC), including anterior cingulate cortex (ACC) and ventral striatum (VS), which were tested using a significance level of *p* < 0.05 (family wise-error (FWE) corrected).

### Psychometric data

Depression, anxiety and pain symptom duration as well as the rating and response data from the PIT task were analysed with analyses of variance (ANOVAs) (two-tailed) with group (patients vs. controls) as intersubjective factor using the Statistical Package for Social Sciences (SPSS) version 15.0 for Windows. We also aimed to determine the role of motivational value in chronic back pain and compared behavioural and brain responses to CSs rated with lower (5–6) versus higher (score > 6) positive valence. We chose a valence rating of 6 for the dividing point to capture really the high levels and have a clear, not intermixed indicator of positive valence.

Moreover, we performed a moderation analysis in the patient sample to test for associations of PIT, pain symptom duration and anxiety and depression. For this moderation analysis, we used brain responses during PIT as dependent (outcome) parameter, pain symptom duration as independent variable and depression and anxiety scores as possible moderators. Whenever the assumption of homogeneity of variances was violated, we applied the Greenhouse–Geisser adjustment and corrected degrees of freedom are reported. For all tests a two-sided Bonferroni-corrected alpha level of 0.05 was employed.

## Supplementary information

Supplementary information
