# Supplementary material for: Brain-behaviour correlates of habitual motivation in chronic back pain
Source: Sci Rep. 2020 Jul 6;10:11090. doi: 10.1038/s41598-020-67386-8 (PMC7338353; doi:10.1038/s41598-020-67386-8)

**Figure 1 Supplement:** Extracted beta values of the hippocampus, amygdala, ventral striatum and anterior cingulate cortex for healthy control and chronic pain patients.

**
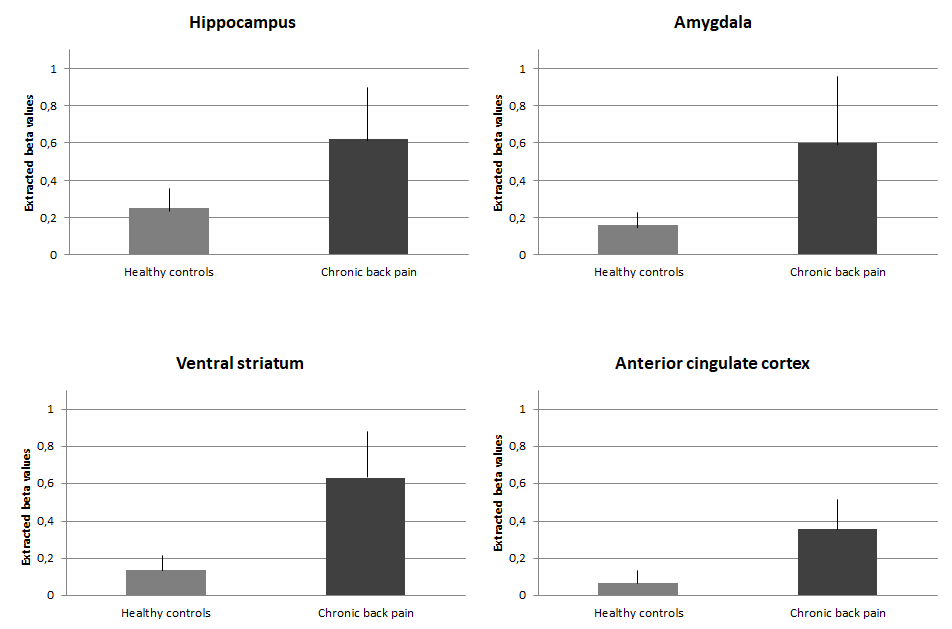
**

**Figure 2 Supplement:** Appetitive Pavlovian-instrumental transfer task.


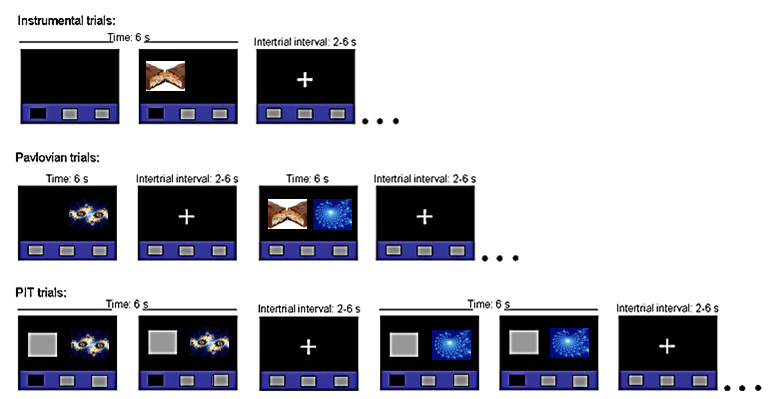

Supplement: Supplementary file 1 — Supplementary information [file 41598_2020_67386_MOESM1_ESM.docx]
